# Antitumor and Antioxidant Activity of S-Methyl Methionine Sulfonium Chloride against Liver Cancer Induced in Wistar Albino Rats by Diethyl Nitrosamine and Carbon Tertrachloride

**DOI:** 10.3390/ijerph18189726

**Published:** 2021-09-15

**Authors:** Tarek Kamal Abouzed, Fayez Althobaiti, Nesreen Adel Abdelkhlek, Ehab Bedir Eldomany, Nasr Elsayed Nasr, Kadry Mohamed Sadek, Samir Ahmed El-Shazly, Khaled A. Kahilo, Doaa Abdallha Dorghamm

**Affiliations:** 1 Biochemistry Department, Faculty of Veterinary Medicine, Kafrelsheikh University, Kafrelsheikh 33516, Egypt; nsr990496@gmail.com (N.A.A.); nasr_157@yahoo.com (N.E.N.); elshazlysamir@yahoo.com (S.A.E.-S.); kahilo2000@yahoo.com (K.A.K.); Doaa_dorgham@yahoo.com (D.A.D.); 2 Biotechnology Department, College of Science, Taif University, Taif 21944, Saudi Arabia; faizh1394@gmail.com; 3 Department of Biotechnology and Life Sciences, Faculty of Postgraduate Studies for Advanced Sciences, Beni-Suef University, Beni-Suef 62511, Egypt; drehabeldomany@psas.bsu.edu.eg; 4 Biochemistry Department, Faculty of Veterinary Medicine, Damanhur University, Damanhur 22511, Egypt; kadry.sadek@vetmed.dmu.edu.eg

**Keywords:** hepatocarcinoma, S-methyl methionine sulfonium chloride, antitumor, antioxidant, anti-inflammatory, anti-metastatic

## Abstract

Liver disease, especially liver cancer, has become a threat facing the world. Now, antioxidant products are garnering great attention for the treatment and prevention of many diseases. S-Methyl methionine sulfonium chloride (MMSC) is a methionine derivative and is present in many vegetables and has anti-inflammatory effects and antioxidants. This is the first study aiming to investigate the antitumor activity of the MMSC. This study was carried out on 60 male Wistar albino rats (4–6 weeks old age) and divided into four groups, with the first group as normal control, second group as hepatocarcinoma induced by diethyl nitrosamine and carbon tetrachloride (DEN/CCL4) group, third group as normal rats treated with MMSC, and fourth group as hepatocellular carcinoma (HCC) induced rats treated with MMSC. Our findings revealed that MMSC administration after HCC induction significantly improved (*p* < 0.05) the liver function biomarkers, including AST, GGT, albumin, globulin, and albumin/globulin ratio (A/G), in comparison with those in the HCC group. Moreover, the histopathological changes of the liver tissue in the HCC group were improved by MMSC treatment. Likewise, the expression levels of tumor necrosis factor-alpha (*TNF-α*), induced nitric oxide synthase (*iNOS*), transforming growth factor (*TGF-1β*), and glypican 3 (*GP3*) were downregulated by MMSC treatment after HCC induction in comparison with those in the HCC-induced group. In conclusion, MMSC showed antitumor activity against HCC induction by DEN/CCl4 through decreasing lipid peroxide formation, the expression level of an inflammatory cytokines such as (*TNF-α*), immunoregulatory cytokines such as (*TGF-1β*), induced nitric oxide synthase, and glypican 3.

## 1. Introduction

Hepatocellular carcinoma (HCC) is one of the leading causes of cancer-related deaths, and the rate of HCC in men is three times higher than in women [1]. In 2013, HCC acted as 4.3% of all cancer cases, and it is counted as the fourth most common cancer in men and eighth most common cancer in women [2]. There are many risk factors for HCC production such as chronic alcohol drinking, aflatoxin B1 infection, hepatitis B and C viral infections [3], diabetes, obesity, and tobacco smoking [4]. In Egypt, aflatoxin B1 contamination and pesticides are thought to play a role in the high prevalence of HCC [5].

There are several carcinogenic agents to the liver; among them is diethylnitrosamine (DEN), which causes hepatic cellular injury through the generation of reactive oxygen species (ROS) and DNA damage [6].

Nitrosamine is considered as an environmental dietary carcinogen because it can be produced as a result of the reaction of nitrite salt, which is used as a meat preservative with the amine group of meat protein [7].

Several studies used diethyl nitrosamine (DEN) to induce hepatocarcinoma in an animal model [8]. DEN treatment alone needs a long time to induce hepatic carcinoma, thus CCL4 is used to promote and enhance the development of liver cancer in a short amount of time [9].

Glypican3 (GP3) is a proteoglycan that belongs to the heparin sulphate (HS) family; it is attached to the cell membrane by a glycosylphosphatidylinositol (GPI) [10]. GP3 expression was normally detected in the liver from the 18th to the 30th week of pregnancy, but no GP3 expression or little expression was found in normal adult liver tissue. Furthermore, the GP3 expression was highly expressed in HCC cells compared with normal liver [11,12,13]. Therefore, GP3 is a markedly useful marker for the diagnosis and prognosis of HCC, thus it may be a potential molecule that is the target of HCC innovative therapies [14]. The role of GP3 in the progression of HCC is unknown. Some studies have suggested that GP3 forms a complex with Wnt via its HS side chain and increases Wnt/catenin signaling in HCC cells [15], and the GP3 cell surface can act as a storage site for heparin-bound growth factors, including fibroblast growth factor (FGF), hepatocyte growth factor (HGF), and heparin-binding epidermal growth factor, all of which may be involved in the development of HCC cells through ERK and/or AKT signaling [16]. A recent study has suggested that high levels of GP3 expression may accelerate HCC cells’ epithelial–mesenchymal transition (EMT) via ERK activation [17], in which EMT has a role in cancer cells’ metastatic behavior and drug resistance [18].

S-Methylmethionine sulfonium chloride (MMSC) is a methionine derivative; is present in many vegetable such as cabbage, kohlrabi, turnip, tomatoes, and celery [19]; and has been called vitamin U [20], although its classification as a vitamin is not accepted. MMSC has antiulcer, antidepressant, and anti-inflammatory properties as well as the capacity to reduce blood lipids. It also possesses wound-healing and cytoprotective properties [21].

The adverse effects of radiotherapy and chemotherapy are increasing the need for scientific researchers to investigate a new strategy as an alternative medicine to improve cancer cell response to treatment. Thus, this study is a novel study to investigate the ameliorative effect of S-methyl methionine sulphonium chloride against HCC-induced albino rats through the reduction of lipid peroxide, an inflammatory cytokine, and GP3 expression.

## 2. Material and Methods

### 2.1. Material

S-Methyl methionine sulphonium chloride was purchased from (Xi’an Realin Biotechnology Company (Xi’an, China)). N-nitrosodiethylamine (NDEA), carbon tetrachloride (CCl4) (anhydrous ≥ 99.50%, product number 289116, Brand Sigma Aldrich), chloroform (product number C2432, Brand SIGALD), and isopropanol (product number I9030, Brand SIGALD) were purchased from Sigma-Aldrich Co. (St. Louis, MO, USA).

Easy-RED^TM^ Total extraction kit (Cat. No. 17063), HiSenScript^TM^ RH [-] cDNA synthesis kit (Cat. No. 25014) was purchased from INTRON Biotechnology, Inc. (Gyeonggi-do, Korea). QuantiTect SYBR Green PCR Kit (Cat. No. 204141) was obtained from QIAGEN (Hilden, Germany). AST (Cat. No. AS 10 61 45), ALT (Cat. No. AT 10 34 45) kits were purchased from Biodiagnostics (Cairo, Egypt). γGT (Cat. No. GGT12460), LDH (Cat. No. LDH117125), Albumin (Cat. No. ALB100250), and total protein (Cat. No. TP116250) kits were purchased from the Biomed diagnostic company (Cairo, Egypt). Lipid peroxides (Cat. No. MD 25 29), catalase (Cat. No. CA 25 17), and SOD (Cat. No. SD 25 21) kits were purchased from Biodiagnostics company (Cairo, Egypt).

### 2.2. Animals

The current research was carried out using sixty male albino rats of average weight (110–150 g), which were obtained from the Egyptian association of biological products and vaccines (Agouza, Giza, Egypt). Rats were housed in well-ventilated plastic cages for one week to acclimate to laboratory conditions (temperature 22–25 °C and 12 light/dark). Throughout the experiment, the animals were given a normal commercial diet (El-Nasr Co, Cairo, Egypt) and water ad libitum. The experiment was carried out in accordance with Kafr-Elsheikh University’s animal care guidelines and the National Science Council’s Guide for the Care and Use of Laboratory Animals (Date No. 16/6/2019).

### 2.3. Preparation of DEN and CCl4

Diethyl nitrosamine was dissolved in physiological saline at a concentration of 0.9%, and then filtrated, and was injected intraperitoneally into the rats at a dose of 200 mg/kg BW for the promotion of HCC [22]. Fourteen days later, carbon tetrachloride solution (CCl4/olive oil; 1:1) was given intraperitoneally at a dose of 1 mL/kg three times per week for 6 weeks [23].

### 2.4. Experimental Design

After the period of acclimatization, the rats were randomly divided equally into four groups, as follows: Group (I): Untreated normal rats received a normal saline (0.5 mL) solution (0.9 % *v*/*v*) intragastrically by stomach tube. Group (II): Rats were treated by DEN/CCl4 to induce the HCC, as previously mentioned. Group (III): Rats were treated with MMSC (50 mg/kg/day) daily by stomach tube. Group (IV): Rats were firstly injected with DEN/CCl4 to induce HCC, followed by MMSC treatment with the same dose for 16 weeks.

### 2.5. Blood Samples

Rats were anaesthetized with an intraperitoneal injection of thiopentone sodium (50 mg/kg body weight) at the end of the experiment [24]. Blood samples were taken from each animal’s retro-orbital venous plexus and stored in clean, dry centrifuge tubes in an ice bath, and centrifuged at 1520× *g* for 10 min at 4 °C for sera separation. The obtained sera was transferred to clean, dry Eppendorf tubes and stored at -80 °C until biochemical parameter analysis.

### 2.6. Tissue Sample

The liver was collected after the decapitation of anesthetized rats. The liver was divided into three parts; the first part (median lobe of the right segment) and the second part (median lobe of the left segment) were taken directly, snap-frozen in liquid nitrogen, and deposited at −80 °C until RNA extraction and real-time PCR, lipid peroxidation, and antioxidant assay, respectively. Meanwhile, the third part (left lateral lobe) was fixed in neutral buffered formalin (10%) for histopathological analysis.

### 2.7. Serum Biochemical Assays

The serum alanine aminotransferase (ALT), aspartate aminotransferase (AST) [25], and LDH [26] activities were measured following the procedures of commercial kits. Furthermore, the serum γGT activity was measured according to the Szasz method [27].

### 2.8. Assessment of Hepaticoxidative/Antioxidant Status

According to the Ohkawa method [28], malondialdehyde (MDA) was determined in the liver tissue homogenate of different groups. Meanwhile, the catalase and superoxide dismutase (SOD) activities were measured using the methods of [29,30], respectively. The liver homogenate was prepared briefly as follows: 1 g of liver tissue was homogenate in 5 ml cold potassium phosphate buffer (100 mM, pH 7), and centrifuged at 1520× *g* for 15 min, then supernatant was collected in a clean, dry Eppendorf tube and preserved at −80 °C until the lipid peroxidation and antioxidant assay.

### 2.9. Histopathological Examination

The liver tissue of different groups was processed and sectioned for histopathology analysis. The procedure can be summarized briefly as follows: after fixation of liver tissue in formalin 10%, the sample was dehydrated in various alcohol concentrations, washed in xylene, and embedded in paraffin blocks. The sectioning was made with 5 µm thickness and was stained with hematoxylin and eosin (H&E) [31]. The digital camera computer interface (Nikon digital camera, Shinagawa-ku, Tokyo, Japan) was used to photograph histological changes in the liver tissue under a light microscope.

### 2.10. Quantitative Real-Time Polymerase Chain Reaction (RT-PCR)

The total RNA was extracted from hepatic tissue of the different groups using the Easy-RED^TM^ Total extraction kit following the manufacturer’s procedure. After dissolving the RNA pellet with RNase-free water, the RNA concentration was assessed using Nanodrop 2000c (Thermo Scientific, Waltham, MA, USA). Single-strand cDNA was synthesized using HiSenScriptTM RH [-] cDNA synthesis kit and following the manufacturer’s procedure. Briefly, 10 μL of the 2X RT reaction solution was mixed with 1 μL of enzyme mix solution, 1 μg of RNA, and completed to 20 μL total volume with RNase free water. The mixture was incubated for 30 min at 50 °C and 10 min at 85 °C. Q RT-PCR analysis was conducted using SYBR green, primers that are listed in Table 1. Primers were synthesized by Macrogen Co. (Seoul, Korea).

The real-time programming was as follows: introductory denaturation at 92 °C/10 min, accompanied by 40 cycles of 92 °C/15 s, 60 °C/30 s, and 72 °C/30 s. The gene expression difference between different groups was measured using the method △△ CT, normalized to β-actin, and expressed as relative mRNA levels compared with placebo.

### 2.11. Data Statistical Analysis

One-way variance analysis (ANOVA) was used to check variances among different experimental groups, along with the Bonferroni test (GraphPad Software Inc., San Diego, CA, USA). The significance of *p* ≤ 0.05 is considered statistically significant. All data were expressed as means + SE.

## 3. Result

### 3.1. Biochemical Findings

**Figure 1:**
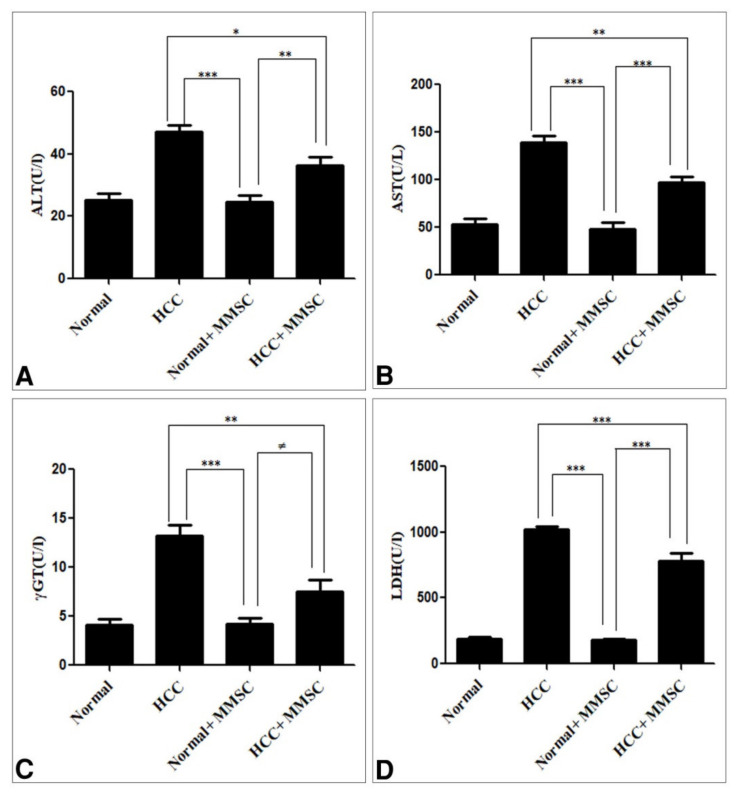
The serum level of (**A**) alanine transaminase (ALT), (**B**) aspartate aminotransferase (AST), (**C**) gamma glutamyltransferase (γGT), and (**D**) lactate dehydrogenase (LDH). Data were presented as mean ± SEM. * Significantly different at *p* < 0.05 using ANOVA followed by Bonferroni’s as a post-hoc test, ** Significantly different at *p* < 0.001, *** Significantly different at *p* < 0.0001, ^≠^ no significantly difference using ANOVA followed by Bonferroni’s as a post-hoc test. Hepatocellular carcinoma (HCC), S-methylmethionine sulfonium chloride (MMSC).

Figure 1 shows the liver biomarkers enzymes, including ALT, AST, GGT, and LDH. These enzymes were significantly increased (*p* ≤ 0.05) in the HCC-induced group in comparison with those in the untreated normal group and MMSC-treated normal group. On the other hand, there were no significant differences in these enzyme activities between the untreated normal group and the MMSC-treated normal group. Interestingly, the MMSC treatment after HCC induction significantly decreased (*p* < 0.05) those enzyme activities and restored nearly up to the basal level, compared with the HCC-only induced group.

**Figure 2:**
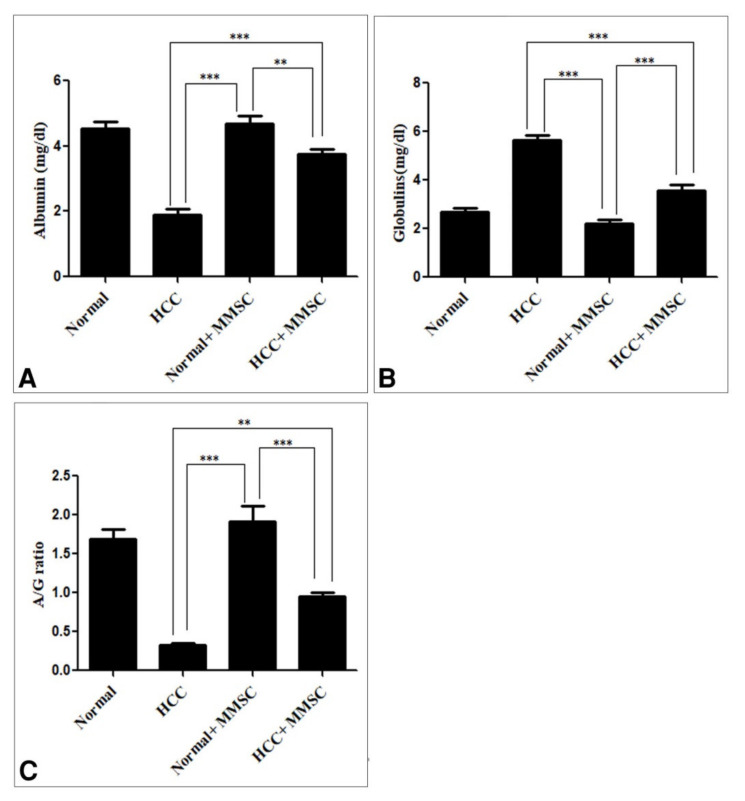
The serum level of (**A**) albumin, (**B**) globulin, and (**C**) the albumin/globulin (A/G) ratio. Data were presented as mean ± SEM. ** Significantly different at *p* < 0.001, *** Significantly different at *p* < 0.0001 using ANOVA followed by Bonferroni’s as a post-hoc test. Hepatocellular carcinoma (HCC), S-methylmethionine sulfonium chloride (MMSC).

The serum albumin level and A/G ratio were significantly decreased (*p* < 0.05) in the HCC-induced group in comparison with those in the normal group and the MMSC normal treated group. Interestingly, the MMSC administration to HCC rats significantly increased (*p* < 0.05) the serum albumin level and A/G ratio compared with the HCC group. Furthermore, there were no significant differences in the serum albumin level and A/G ratio between the untreated normal group and the MMSC-treated normal group, as shown in Figure 2A,C. In contrast, the serum globulin was significantly elevated (*p* < 0.05) in the HCC group compared with that in the untreated normal group and MMSC-treated normal group. Meanwhile, the MMSC treatment after HCC induction significantly decreased (*p* < 0.05) the serum globulin and restored nearly up to the basal level in comparison with that in the HCC-induced group, as shown in Figure 2B.

### 3.2. Oxidative/Antioxidant Activity

**Figure 3:**
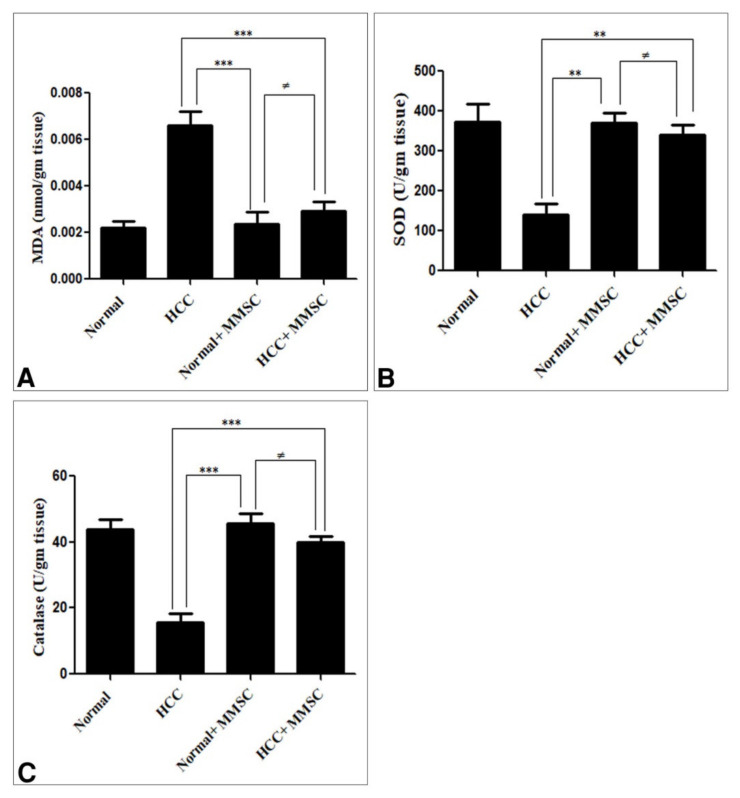
Serum level of (**A**) malondialdehyde (MDA), (**B**) superoxide dismutase (SOD), and (**C**) catalase in all groups. Data were presented as mean ± SEM. ** Significantly different at *p* < 0.001, *** Significantly different at *p* < 0.0001, ^≠^ no significantly difference using ANOVA followed by Bonferroni’s as a post-hoc test. Hepatocellular carcinoma (HCC), S-methylmethionine sulfonium chloride (MMSC).

The hepatic MDA activity was significantly increased (*p* < 0.05). Moreover, the hepatic activities of SOD and CAT were significantly decreased (*p* < 0.05) in the HCC induction group in comparison with those in the untreated normal group and MMSC-treated normal group. Likewise, the MMSC administration after the HCC induction group significantly decreased (*p* < 0.05) the MDA activity, increased the SOD and CAT activity, and restored nearly up to the basal activity, compared with the HCC-only induced group. There were no significant differences between MDA, SOD, and CAT activities for the MMSC-treated normal group and the untreated normal group (Figure 3).

### 3.3. Histopathogy Findings

**Figure 4:**
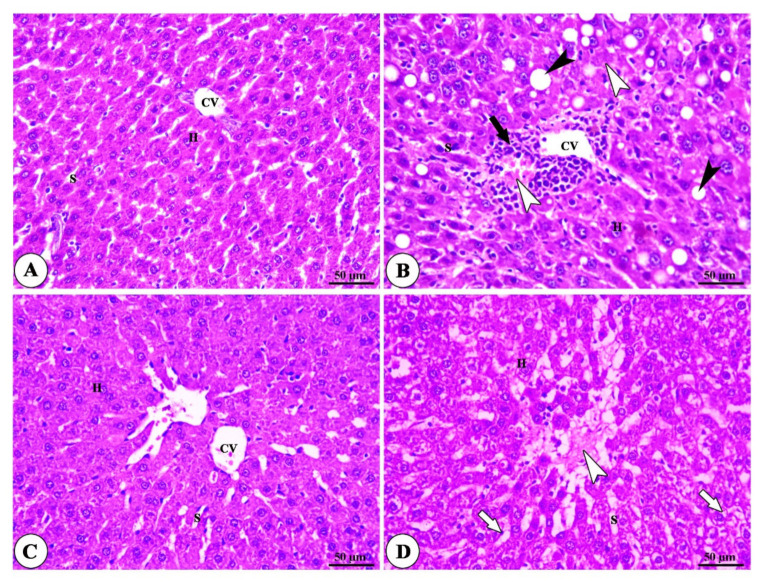
Histomicrograph of liver of normal, hepatocellular carcinoma (HCC), S-methylmethionine sulfonium chloride (MMSC) alone, and HCC + MMSC groups. (**A**,**C**) show polyhedral-shaped hepatocytes (H) separated by blood sinusoids (S) that appear to have a normal architecture. (**B**) shows the steatohepatic variant of HCC (black arrow head) with necrosis of hepatocytes (white arrow head) and inflammatory cells infiltration (black arrows). (**D**) shows the focal area of hepatic necrosis (white arrow head) and microsteatosis of hepatocytes (white arrows) with dilated and congested in MMSC group and central vein (CV). H&E.

The examination of histopathology photography has revealed the normal structure of liver tissue in the normal group and the MMSC-treated normal group, as shown in Figure 4A,C. The liver of the animals treated with DEN/CCl4 showed neoplastic changes, including polymorphisms and clear cytoplasmic pressure atrophy to the adjacent hepatocytes (Figure 4B). Moreover, the MMSC treatment after HCC induction exhibited mild necrosis of the hepatocyte, mild dilatation of blood sinusoid, the absence of tumor lesions, mild vascular degeneration, and hepatic apoptosis (Figure 4D).

### 3.4. Inflammatory Cytokines, and Immunoregulatory Cytokines Expression by qRT-PCR

**Figure 5:**
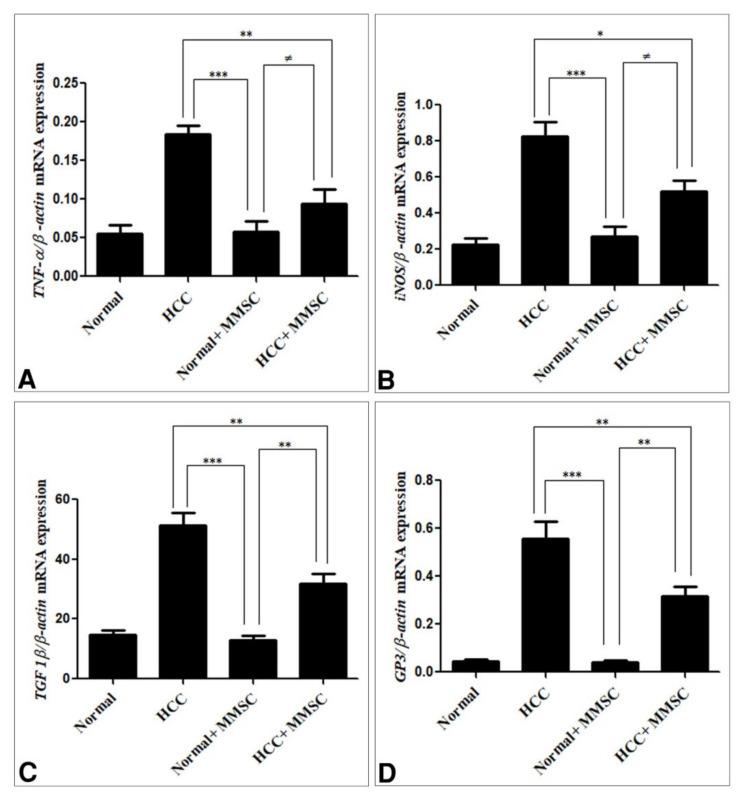
mRNA expression of genes (**A**) TNF-α, (**B**) iNOS, (**C**) TGF-β1, and (**D**) GP3 in liver tissues. The mRNA expression levels were measured by qRT-PCR and normalized to β-actin binding protein. The following primer sets were used for the detection (Table 1). Data are the mean ± SEM. * Significantly different at *p* < 0.05 using ANOVA followed by Bonferroni’s as a post-hoc test, ** Significantly different at *p* < 0.001, *** Significantly different at *p* < 0.0001, ^≠^ no significantly difference using ANOVA followed by Bonferroni’s as a post-hoc test. Hepatocellular carcinoma (HCC), S-methylmethionine sulfonium chloride (MMSC).

The antitumor activity of the S-methylmethionine sulfonium chloride was confirmed through investigation of its effect on the expression of inflammatory cytokine (*TNF-α*, *iNOS*, *TGF-1β*) and *GP3* by qRT-PCR. This present study has shown that the expression level of these inflammatory cytokines and GP3 was significantly up-regulated (*p* < 0.05) in the HCC-induced group in comparison with those in the normal and MMSC normal treated group Furthermore, the MMSC treatment for the HCC-induced group significantly down-regulated (*p* < 0.05) the expression level of these genes compared with those in the HCC-induced group. In addition, the expression level of these genes had no significant differences between normal and MMSC normal treated group, as shown in Figure 5.

## 4. Discussion

In some patients, the liver tumor cannot be removed, does not respond to chemotherapy, only moderately respond to radiotherapy, and leaves patients with limited treatment alternatives [32]. Because of the limited treatment options for hepatocellular carcinoma, several natural compounds and their secondary metabolites are being studied to see if they have a preventive or anticancer effect [33]. DEN is a strong carcinogenic substance, which can cause changes in the nucleic acid repair system and lead to oxidative stress [34]. CCl4 is a tumor promotor for enhancing the induction of HCC in a short amount of time in an animal model [9]. According to the previous studies, DEN and CCl4 are usually used to induce HCC in albino rats. In this study, the possible hypothesis is the antitumor activity of MMSC through the ability to reduce lipid peroxide and increase antioxidant activity and decrease the expression of GP3. Our result confirmed this hypothesis, in which the MMSC administration ameliorated the histopathological changes, liver function biomarkers, oxidative damage, the expression level of the inflammatory cytokine, and GP3, which was induced by the DEN/CCl4 injection to induce HCC in rats. Many previous studies have been documented where in injection of the DEN/CCl4 increased hepatic enzymes such as ALT, AST, and LDH, and indicated hepatic damage [35]. Moreover, the increased LDH activity indicates the hepatic cell membrane damage and loss of intracellular enzyme and induces cell death. In parallel, our findings have shown significant alterations in the activity of liver function markers including ALT, AST, GGT, albumin, globulin, and the A/G ratio in the HCC-induced group in comparison with those in the normal group. Interestingly, the MMSC treatment restored the alterations of the liver biomarkers such as ALT, AST, GGT, LDH, albumin, globulin, and the A/G ratio to near normal activity, consistent with another study [36] reporting that S-methylmethionine sulfonium treatment decreased the ALT and AST level, and ameliorated the liver injury of amiodarone-administered rats. Our findings also agree with another study [37], which reported that S-methyl methionine sulphonium chloride can restore the level of liver biomarkers including ALT, AST, and ALP in valproic acid-induced liver injury in rats. This may be owing to possibly maintaining the membrane integrity of liver cells by the MMSC treatment, and this is an indicator of the possible hepatoprotective effect of the MMSC against HCC-induced rats.

DEN reportedly produces lipid peroxidation (LPO) products, such as malondialdehyde and 4-hydroxynonenal, which can interact with various molecules, and causes oxidative stress and carcinogenicity; further, this reaction can also contribute to an increase in free radical generation, which can decline cellular antioxidant defenses [38]. Inconsistent with our result, elevation of MDA activity and reduction of SOD and catalase activity in the HCC-induced group have been shown. In contrast, the MMSC treatment decreases the MDA level, and increases the antioxidant activity including SOD and catalase. Our findings are in agreement with another previous study, which reported that MMSC treatment increased the activity of catalase, SOD, GPx, and GST in liver toxicity induced by ameliorating in rats [36]. This confirms the protective role of MMSC against HCC induced in rats, through restoring the oxidative and antioxidant status. Furthermore, the hepatoprotective and antitumor activity of MMSC was confirmed by the histopathological analysis, which has shown improvement in the neoplastic lesion, necrosis, and hepatocyte degeneration induced in HCC rats, and this study is the first to report that MMSC plays antitumor and hepatoprotective roles. The cytokines are the hormones of the immune system that play important roles in tumor initiation, maintenance, and progression [39]. *TNF-α*, *IL-6*, *IL-10*, and *TGF-β* are secreted by neoplastic cells and tumor-associated macrophages [40]. This study reported that the expression level of *TNF-α*, *TGF-β*, and *iNOS* was significantly elevated in the HCC-induced group. The MMSC treatment decreased the expression of these genes. These findings are in agreement with another study that documented that MMSC has anti-inflammatory, anti-ulcer, and antioxidant properties [36]. In addition, Glypican 3 (GP3) is highly related to the appearance and development of liver cancer, is used for diagnosis, and is an important target for HCC immunotherapy [41]. Our finding revealed that the expression level of GP3 was increased in HCC-induced rats, in agreement with another study [42], which documented an increased serum level of GP3 level in DEN-treated rats. All biochemical, pathological, and molecular analysis results of this study indicate the antitumor and hepatoprotective effects of the S-methyl methionine sulphonium chloride.

This study indicated the possible mechanism of antitumor activity of MMSC against HCC induction. The antitumor activity of MMSC may be due to its ability to reduce the lipid peroxide formation and its antioxidant activity, in addition to its ability to ameliorate the expression of inflammatory, immunoregulatory cytokines, and Glypican 3 genes. In addition, this mechanism could be predicted based on the MMSC metabolism and its parent compounds, where MMSC is a derivative of methionine, which is involved in many biosynthetic pathways as a main source of the sulfonium functional group. It is biosynthesized from L-methionine, which is first converted to S-adenosylmethionine (SAM), and then SAM is converted to MMSC through replacement of the adenosyl group by a methyl group. The conversion is catalyzed by the enzyme methionine S-methyltransferase [43]. Interestingly, SAM synthesis is inhibited in chronic liver diseases; therefore, SAM plays a pivotal hepatoprotective role. Consequently, the reduced SAM level is reported as a key factor in liver cirrhosis and subsequent HCC [44]. In contrast, exogenous SAM can inhibit HCC development by recovering the normal level of liver SAM [45].

## 5. Conclusions

The biochemical, histopathoglogical, and molecular results indicate that S-methyl methionine sulfonium chloride has anti-tumor properties and could be a useful treatment of liver cancer induced by DEN/CCl4 in rats through the anti-oxidative system and decreasing the expression of proinflammatory cytokines and the GP3 gene.

## Figures and Tables

**Table 1:** Sequence of primers for PCR.

	Sense	Antisense	Annealing Temperature
TNF-α	GACCCTCACACTCAGATCATCTTCT	TTGTCTTTGAGATCCATGCCATT	60
iNOS	TCTTCAAGGACCTACCTCAGGC	GCTAAGGCAAAGCTGCTAGGTC	60
TGF B-1	TCACTTGTTTTGGTGGATGC	TTCTGTCTCTCAAGTCCCCC	60
GP3	GTGCTGGAACGGACAAGAG	TTCTTCATCCCATTCCTTGC	60
β-actin	TGTTGTCCCTGTATGCCTCT	TAATGTCACGCACGATTTCC	60

## Data Availability

Data are available upon request.
